# Atomic-scale configurations of synchroshear-induced deformation twins in the ionic MnS crystal

**DOI:** 10.1038/srep05118

**Published:** 2014-05-30

**Authors:** Y. T. Zhou, Y. B. Xue, D. Chen, Y. J. Wang, B. Zhang, X. L. Ma

**Affiliations:** 1 Shenyang National Laboratory for Materials Science, Institute of Metal Research, Chinese Academy of Sciences, Wenhua Road 72, 110016 Shenyang, China

## Abstract

Deformation twinning was thought as impossible in ionic compounds with rock-salt structure due to the charge effect on {111} planes. Here we report the presence and formation mechanism of deformation {111} twins in the rock-salt manganese sulphide (MnS) inclusions embedded in a hot-rolled stainless steel. Based on the atomic-scale mapping under aberration-corrected scanning transmission electron microscopy, a dislocation-based mechanism involved two synchronized shear on adjacent atomic layers is proposed to describe the dislocation glide and consequently twinning formation. First-principles calculations of the energy barriers for twinning formation in MnS and comparing with that of PbS and MgO indicate the distinct dislocation glide scheme and deformation behaviors for the rock-salt compounds with different ionicities. This study may improve our understanding of the deformation mechanisms of rock-salt crystals and other ionic compounds.

Twinning is an important deformation mechanism in metals and intermetallic compounds^1^. Under certain deformation conditions, deformation twins have also been identified in semiconductors^2^ and some other ceramics like alumina^3^ and boron carbide^4^. Since the charge effect dominates the deformation behavior in ionic crystals, the deformation behaviors of rock-salt ionic compounds whose lattice mechanics were relatively well know have attracted much attentions ever since dislocation theories were proposed^5^,^6^,^7^,^8^,^9^,^10^,^11^,^12^,^13^,^14^. However, deformation twinning in such compounds has been rarely reported in experiments because of the charge effect that is inhibiting dislocation activation on twin planes. In rock-salt ionic crystals, the most close-packed {111} planes contain ions of only one charge sign on either layer, which would have strong Coulomb interaction^6^. Thus, slip on {111} planes was not observed except in the ionic compounds with weak ionicity (e.g. PbS)^11^,^12^. Compared with slip, twinning occurs by partial dislocations gliding on successive {111} planes and therefore was thought as ‘unlikely'^15^.

It is worth noting that the glide scheme of dislocation may significantly influence the slip and twinning formation. Atomic–scale observation is needed to clarify these defects and better understand the formation mechanism. The recent progress in aberration-corrected scanning transmission electron microcopy (STEM) provides opportunity to directly visualize atomic details at a sub-angstrom resolution. For instance, the dislocation core of Cr_2_Hf was investigated by aberration-corrected scanning transmission electron microscopy (STEM) and a dislocation scheme consisting of two shears on adjacent atomic layers has been proposed to explain both deformation and phase transformations^16^. In ionic crystals, the dislocation-based deformation may become more complex and interesting because of the Coulomb interaction between ions^17^.

Here, we report the observation of deformation twins in rock-salt manganese sulphide (α-MnS) embedded in a stainless steel. Both aberration-corrected STEM observation and first-principles calculations indicated that the synchronized motions of atoms in neighboring atomic layers can effectively reduce the energy barrier for twin dislocation glide and facilitate the twin formation. Since the sulphide inclusions were generally considered to play important roles in steel mechanical properties^18^,^19^,^20^,^21^, study on the structure and formation conditions of deformation twin in MnS is believed to be helpful for understanding its relevance to the mechanical properties of alloys. More importantly, the knowledge concluded from the twin dislocations on polarized planes in rock-salt structure may shed a light on our understanding of deformation behaviors of other ionic crystals.

## Results

### The atomic structure of twin boundary

During the process of hot-rolling (800–1200°C) of stainless steels, MnS inclusion is remarkably deformed and developed into an elongated shape. The needle-shaped MnS inclusions are parallel to the rolling direction. The MnS in the present study has a rock-salt structure with lattice parameter of *a* = 0.52 nm.

**Figure 1:**
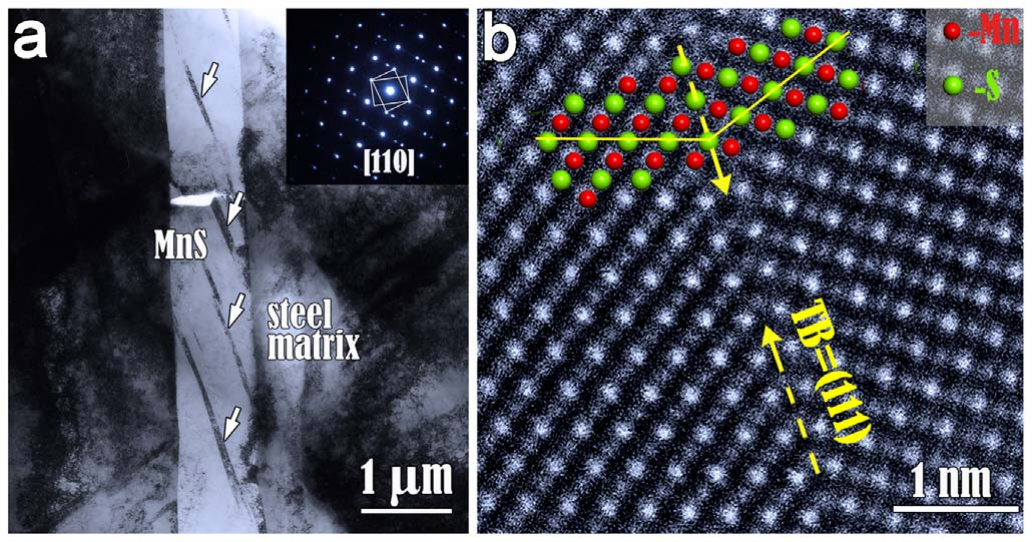
The morphology of deformation twins in a MnS inclusion embedded in a stainless steel and an atomic resolution HAADF image of a twin boundary. (a) Low magnified TEM image and a corresponding SAD pattern showing the presence of deformation twins in MnS inclusion. The twin plane is identified as (111) plane. (b) A high resolution HADDF image of a twin boundary viewed along 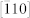 direction. The atomic model of TB is superimposed, where the manganese ions and sulphur ions are represented by red and green spheres, respectively. The atomic configuration of the twin boundary was identified to be the mono-layer of S instead of Mn.

In some MnS inclusions, several parallel twin lamellas are found. As shown in Fig. 1a, several thin twins are clearly visible in a MnS inclusion section (viewed near edge on and oriented along the 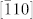 zone axis). Usually, a growth twin has a perfect twin boundary. In contrast, the twin boundaries shown in the image are not straight and contain distinct defects, which indicate that the twins in MnS inclusions should be classified as deformation twin. The inset in Fig. 1a is a selected-area electron diffraction (SAED) pattern, in which the twin relationships are represented by two rectangles connecting with diffraction spots from twins and matrix. The twin plane is (111) plane, which is the most common twin plane in the fcc structure and consistent with the reports of growth twins in silver halides^22^,^23^.

Since the lattice MnS consists of a cation and an anion as a basis, here we denote the stacking sequence of the (111) planes as AcBaCbAcBa… where A, B, C = Mn and a, b, c = S. As a result, there will be two possible arrangements for a twin, for example, AcBaCbAbCaBcA… or AcBaCbCaBcA… where A and b denote the twin plane. These two arrangements differ in the choice of the atomic layer as the twin plane. To provide insight into the structures of the twin boundary in MnS, high angle annular dark field (HAADF) Z-contrast STEM technique were performed, which displays strong contrast associated with the atomic number ‘Z' of the local composite^24^.

Fig. 1b shows a HADDF image of the twin boundary. The brighter dots in the atomic resolution STEM image correspond to the manganese columns, and the weaker ones are sulphur columns. It is clearly seen from the image that the twinning boundary lies in a mono-layer of sulfur. An atomic model along 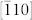 direction is superimposed. The cations and anions are represented by red and green spheres, respectively. The stability of the twin structure in MnS was verified by the DFT calculations on the twin boundary energy (TBE) and electronic structure^26^.

### Twinning formation in MnS

**Figure 2:**
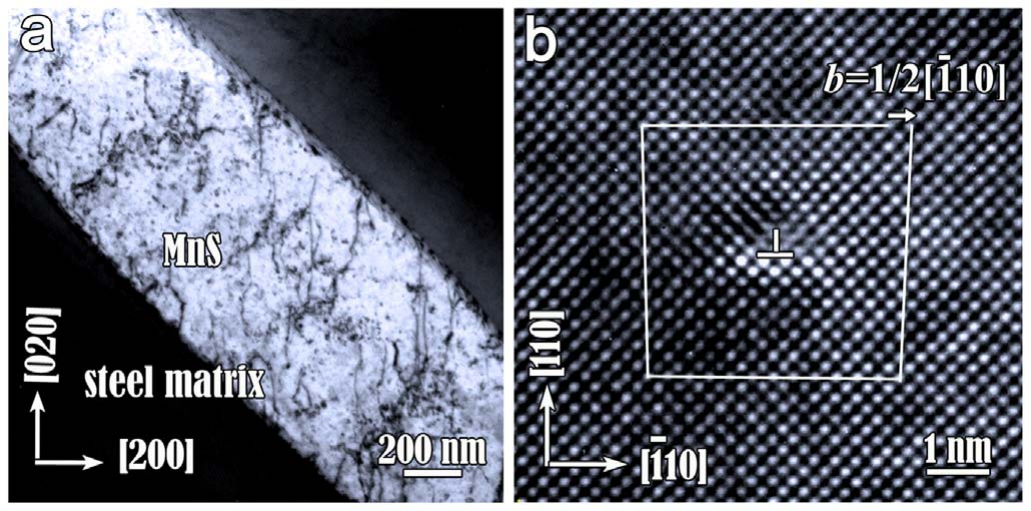
TEM images of MnS inclusion without deformation twin. (a) A bright-field TEM image showing the plastic deformation of MnS is achieved by cross slip. Incident beam direction is close to [001]. g = 020. (b) The HRTEM image of a egde-on dislocation with Burgers vector (a/2) 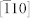 and slip plane [110].

It is known that the primary slip system in rock-salt ionic crystals is {110}<110>. This is the case in MnS^25^, which is confirmed by our TEM experiments. Fig. 2a is a TEM image of the MnS inclusion without twin. The long axis of MnS is close to [110] direction. Distinct dislocation contrast in MnS indicates that the inclusion was plastically deformed by cross-slip in such case. A high-resolution TEM image of the dislocation is displayed in Fig. 2b. By drawing a Burgers circuit surrounding the dislocation core, the Burgers vector and slip plane of the dislocation are determined as (a/2) 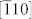 and (110), respectively.

**Figure 3:**
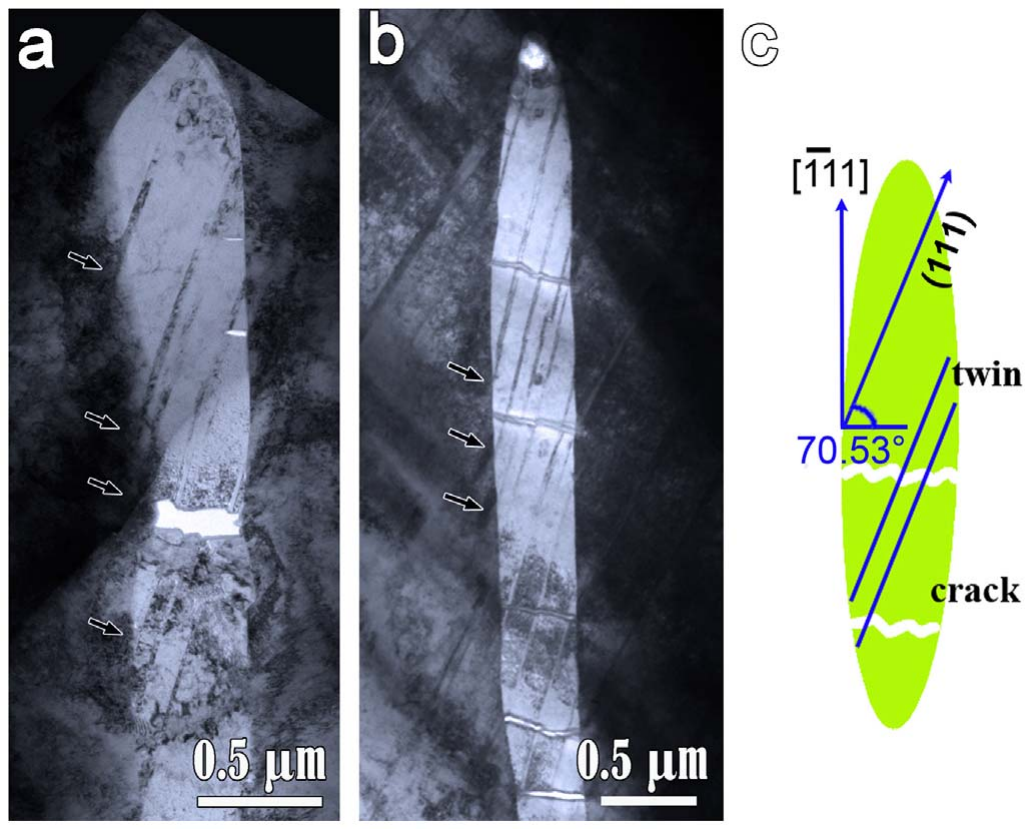
TEM analysis and schematic illustration of the loading conditions for twinning in MnS (a) and (b) Typical twins-containing MnS sections in the steel sample. The black arrows denote the local high stress at the MnS/steel interfaces. (c) Schematic illustration of the deforming conditions for twin nucleation in MnS inclusions in the present stainless steel sam.

Deformation twins in MnS inclusion are not prevalent, which suggests the twin formation should be subjected to certain stress conditions. Fig. 3a and b show typical morphologies of deformation twins in MnS. It is seen that almost all of the twin lamellas in MnS inclusion have a certain included angle i.e. ~20° with the long axis of MnS, namely, the rolling direction. It can be explained by the orientation relationship between crystal orientation and deformation direction (see Fig. 3c). We assume that during the deformation the force along the rolling direction is dominated. When the crystal is deformed by uniaxial tension along 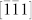 direction, the Schmid factor for {110}<110> slip is zero. Then the partial dislocations on {111} planes nucleates, and as a result, twinning happens. The included angle between (111) and 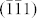 planes is 71.53° which is exactly in agreement with the present observations.

Moreover, the twin lamellas almost initiate from the MnS/steel interface. As arrowed in Fig. 3a and b, it always features obvious contrast of high density of dislocations in metal matrix near the twin heads. Hence, the high strain concentration localized at the MnS/steel interface might be responsible for the nucleation of the twin dislocations.

**Figure 4:**
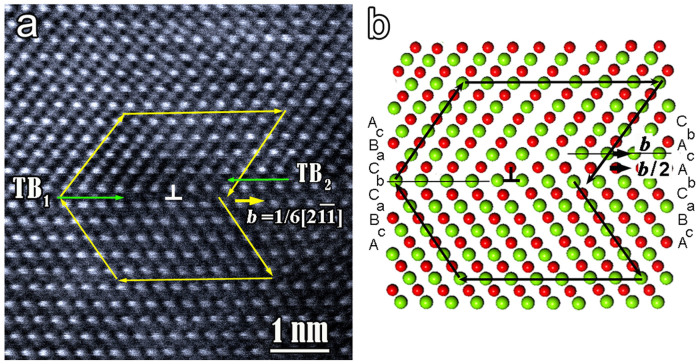
High resolution HAADF image and the corresponding atomic model of a twin dislocation in MnS (a) HR-STEM image of a twin dislocation with Burgers vector (a/6) 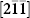, the blank arrows indicate the positions of Mn columns at the dislocation core; (b) a corresponding atomic model showing that the glide of twin dislocation involves synchronized shears on two successive atomic layers. The original twin sequence is assumed to be AcBaCbCaBcAb… Passage of a twin dislocation generates the new twin sequence AcBaCbAcAbCa….

When a partial dislocation moves over the twin plane, it may create a perfect new twin boundary on the neighboring {111} planes and leave a step where it stops. The atomic structure of the step should reflect the glide scheme of partial dislocation to form a twin. Figure 4a shows a STEM image of a coherent twin boundary with a dislocation lying on the twin plane. The corresponding atomic model is shown in Fig. 4b. The step contains two atomic layers so the twin boundary (TB_1_) moves to the neighboring sulphur layer (TB_2_). Carefully analyzing the configuration of twin dislocation indicates that although it has a total Burgers vector **b** = (a/6)<112> (where a is the lattice constant) as the case in fcc metals, it glides involving two synchronized shears in different directions on the adjacent atomic layers. If TB_1_ is assumed to be the original twin boundary, the Mn atoms in the layer ‘C' above the original twin plane TB_1_ move by **b**/2 to position ‘A', implying the motion vector should have 30° angle with viewing direction. Relative to the Mn layer, the upper half-crystal above the Mn layer ‘C' moves by **b** as a rigid body. As a result of the synchronized motion of atoms, a new coherent twin boundary forms on the adjacent sulphur plane.

## Discussion

Several dislocation-based mechanisms^27^,^28^,^29^,^30^,^31^ have been proposed to explain the nucleation of twin. Although the sources of twin dislocation emission are different between each model, it is widely accepted that the partial dislocation gliding on successive planes to form a multi-layer stacking fault, namely, twin. The formation of stacking fault and twin formation in rock-salt structure was proposed earlier based on crystallographic glide^9^,^15^. An intrinsic fault on {111} planes was assumed to form via one half of a crystal above the slip plane sliding by (a/6)<112>, relative to the other, resting half-crystal. Such process was called crystallographic glide, which is a general form for slip in monometallic crystals. The stacking sequence of an intrinsic fault was denoted as AcBaCb⋮**·**CbAcBa… The vertical dot line in the above sequences marks the stacking fault. Successive crystallographic glide on every neighboring atomic layer may result in a new sequence AcBaCbCaBcA… i.e. the twin structure.

**Figure 5:**
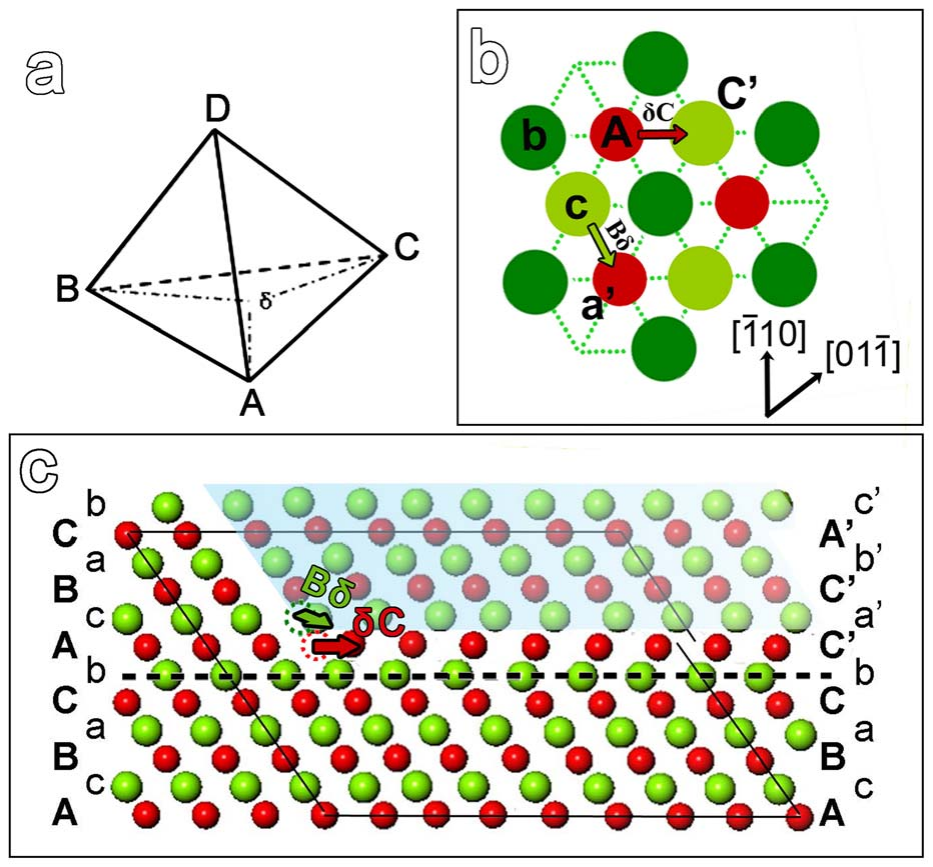
A schematic model of the synchroshear process for dislocation motion in MnS. (a) a Thompson's tetrahedron, (b) a schematic plan view of the S–Mn–S triple layer in MnS and (c) 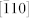 projection of a partial dislocation connected with a stacking fault. The crystal on the left of the dislocation is in a perfect stacking sequence, while the right part shows a stacking fault generated by synchroshear. The arrows indicate the moving direction of the atoms from initial positions (denoted by blank dotted circles) to the final positions.

However, our observation indicates that the twin dislocation glide in MnS is the synchroshear process^16^ which involves two shears in different directions on adjacent atomic layers. In order to visualize the atoms motion during the process, a model of a stacking fault will be helpful. A Thompson's tetrahedron, a schematic top view of the S–Mn–S layers and the 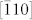 projection of a partial dislocation connecting with a stacking fault are shown respectively in Fig. 5. The stacking fault resultant from atoms motion is illuminated in a top (Fig. 5b) and side view (Fig. 5c), respectively. The half-crystal under slip plane is assumed to be fixed, then another half-crystal above manganese layer ‘A' which is covered by shadow in Fig. 5c moves in the direction of Bδ. At the same time, additional shears of the atoms in middle layer ‘A' operates in a direction of either δC or δA. Arrows in the images represent the moving directions and distances of the atoms in both layer ‘c' and ‘A' from their initial sites (denoted by dotted blank circles) to the final states. The synchronized motion creates the stacking fault with sequence AcBaCb⋮**·**CaCb…. Passage of two or more synchro-Shockley dislocations on the neighboring planes successively results in a twin sequence AcBaCbCaBcA….

To explore the glide scheme of the dislocation on {111} planes in MnS, DFT calculations of the generalized planar fault energy (GPFE) curves^32^,^33^,^34^ have been performed. The GPFE curves are dotted by using the total energies of the supercells for a chain of intermediate configurations along the deformation paths, which determine the energy barriers that twin dislocations must overcome to nucleate a stacking fault and a twin. During a transformation process, the shear parameter s is defined as the relative displacement of two half-parts of a crystal through rigid shift along the deformation path. The energy data when s = 0 corresponds to that of bulk MnS crystal; the maximum, γ_us_, appears at s = 0.5 and gives the energy barrier of nucleation of a partial, which is proportional to the critical stress for the emission of a partial dislocation; and when s = 1, γ_isf_ corresponds to the energy of intrinsic fault generated by a crystallographic glide (SF-I) or a synchroshear (SF-II).

**Figure 6:**
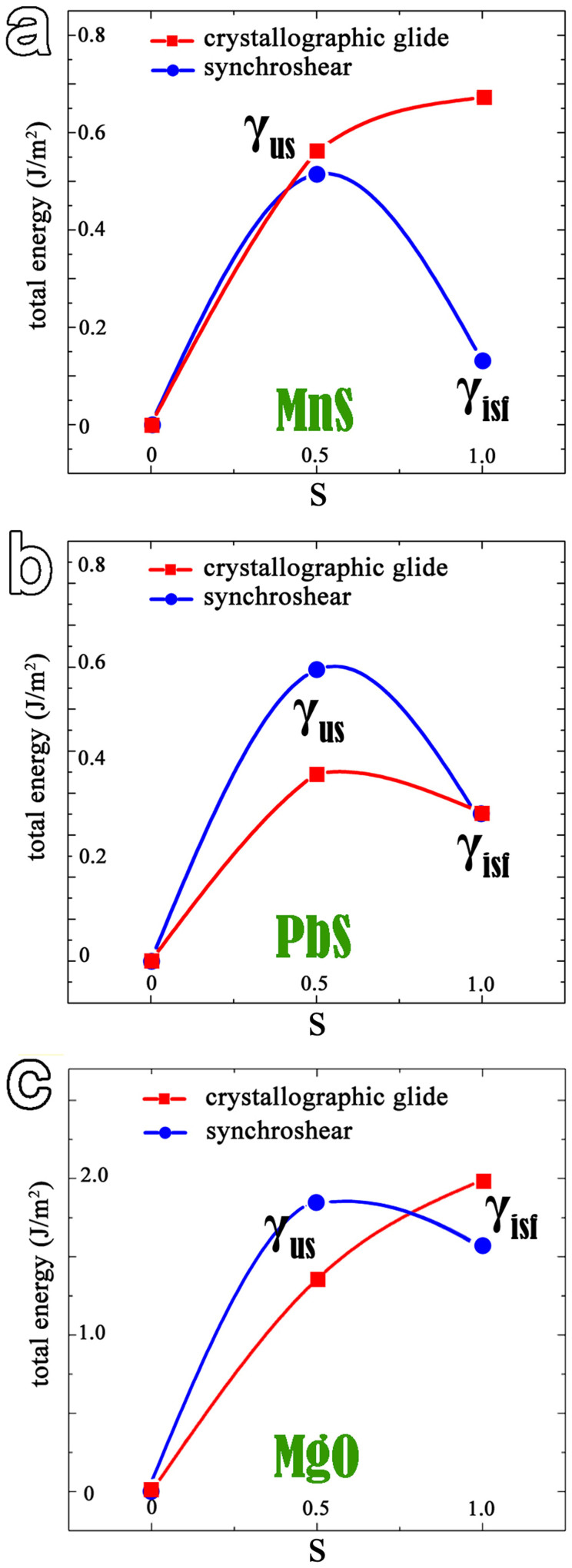
Energy barriers for a partial dislocation nucleation by crystallographic glide and synchroshear in the MnS, PbS and MgO compounds. In the energy profiles, the zero point at the total-energy axis marks the total energy of the bulk crystal. The discrete data points correspond to the calculated results. The continuous curves were fitted by spline functions. It is seen that the synchroshear mechanism is energetically favored for dislocation nucleation on {111} planes in MnS. While the pathway of crystallographic glide is an energy-rising process. The case in PbS, however, is the opposite. Due to the strong ionic bonds in MgO, relative slide between adjacent {111} planes is rather difficult.

In Fig. 6a, the upper red fitting curve shows the energy barrier to form a final fault by a crystallographic glide. The results demonstrate that the pathway is an energy-rising process and the energy of SF-I is about 0.67 J/m^2^. The lower (blue) curve with circles shows the energy profile for a synchroshear process. It indicates that the deformation pathway of synchroshear is energetically favored due to the lower energy barrier (0.51 J/m^2^). Most importantly, the energy of the final state after synchroshear, i.e. SF-II, is about five times lower than that of SF-I. In addition, by analyzing the atomic and electronic configuration of SF-I and SF-II (not shown), the higher energy of SF-I should ascribe to that the S ions in the next-neighboring fault layers are in the same stacking position, which may lead to more superposition of S-S electronic density. As a result, the electrostatic force, as well as the total energy could be further enhanced. Therefore, the partial dislocation gliding by synchroshear can facilitate the stacking fault/twinning formation in MnS.

In ionic compounds, the larger electronegativity difference between cation and anion represents the stronger ionicity. The difference value of MnS (Δχ_MnS_ = χ_S_ − χ_Mn_ = 1.03) falls in the value range between that of MgO (Δχ_MgO_ = 2.13) and PbS (Δχ_PbS_ = 0.23). To obtain a full picture of the dislocation glide scheme on {111} planes in rock-salt ionic crystals, a comparative study of MnS, PbS and MgO is carried out by DFT calculations.

The diagram of energy versus shear parameter of PbS is drawn in Fig. 6b. Although the total energies of two types of stacking fault configurations (SF-I and SF-II) in PbS are almost same, the energy barrier (0.69 J/m^2^) for synchroshear is much larger than that (0.45 J/m^2^) for crystallographic glide. Hence in PbS crystal the crystallographic glide should be more favorable than synchroshear. Figure 6c shows the energy to nucleate a partial dislocation in MgO. Since the strong Mg-O ionic bonds hinder the relative sliding between layers, the energy barrier required for slip on {111} planes in MgO is rather large and cannot access easily in either glide scheme. In Fig. 6, it can be seen that the energy barrier required to nucleate a partial dislocation in a crystal is remarkably increased with increasing ionicities (such a sequence as PbS, MnS and MgO). Sychroshear process is a compromised pattern to overcome the electronic interaction between ionic layers in the MnS crystal with medium ionicity.

**Figure 7:**
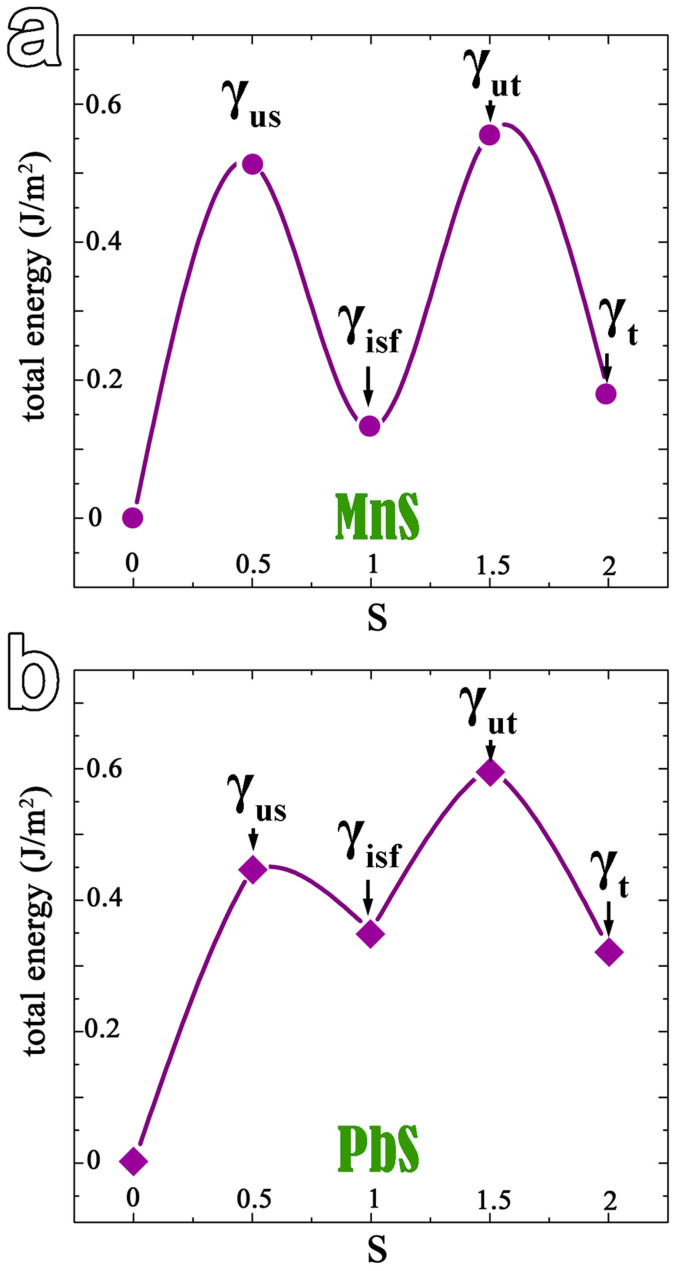
GPFE curves for MnS and PbS. (a) The diagrams of energy versus shear parameter of MnS and (b) for PbS. The relative higher stacking fault energy and lower γ_us_/γ_ut_ value for PbS, compared with that for MnS indicate that cross-slip and twinning is favored in PbS crystal and in MnS crystal, respectively.

According to the above discussion, the pathways to create faults in MnS and PbS may be chosen as synchroshear and crystallographic glide, respectively. To compare the different deformation behaviors in MnS and PbS, the expanded GPFE profiles of MnS and PbS are displayed in Figs. 7a and 7b, respectively. The energy associated with nucleation a twin dislocation can be determined by unstable twin fault energy γ_ut_. In curves, the minimums γ_isf_ and γ_t_ are the intrinsic fault energy and twin energy. Then, the competition between deformation through the motion of a full dislocation and twinning depends on the ratio γ_us_/γ_ut_^34^. The larger the value of γ_us_/γ_ut_, the higher twinning formability will have for a crystal. In Fig. 7, the γ_us_/γ_ut_ value for MnS is near 0.92, while for PbS is 0.74. This means that the twinning formability of MnS is higher than that of PbS, which are consistent with the experimental observations on MnS and PbS^11^,^12^.

In summary, we have studied the structure and formation mechanism of the twin in MnS inclusion embedded in a 316F stainless steel by means of high resolution TEM. By analyzing the twin dislocation, the synchroshear mechanism is applied to describe the twin dislocation glide and consequently twinning formability. The DFT calculations demonstrated this kind of dislocation scheme is energetically favorable. As we know, the dislocation structure and dynamic are crystallography-dependent^35^, the comparative study of MnS, PbS and MgO indicates that the ionicity plays a critical role in the dislocation glide schemes and deformation behaviors even in the compounds with same crystallographic structure.

## Methods

A commercial 316F stainless steel with high-sulphur content (0.16%) was chosen because it can provide a large number of MnS inclusions for analysis. The steel was made by Nippon Steel and Sumikin Stainless Steel Corporation. It was hot-rolled into rods with diameter of 1 cm. Such a rolling made the MnS inclusions needle-shaped. TEM samples parallel to the rolling direction were sliced by a linear precision saw. The samples were then ground using variant grit silicon carbide papers, polished with diamond paste to 1 μm finish, and finally thinned by ion-milling.

The TEM observations were performed in a Tecnai F30 microscope. Atomic resolution high-angle annular dark field (HAADF) scanning transmission electron microscopy (STEM) images were recorded in a Titan G^2^ 60–300 microscope with double aberration (Cs) correctors. The convergent semiangle was chosen as 20.8 mrad, and a large inner collection angle was set as 50 mrad.

All calculations were carried out with the Vienna Ab initio Simulation Package (VASP)^36^,^37^ in the framework of density functional theory (DFT). We adopted the projector-augmented wave (PAW) method^38^,^39^ to describe the core-valence electron interaction and the generalized gradient approximation (GGA) formulated by Perdew, Burke and Ernzerhof (PBE)^40^ to treat the exchange correlation between electrons. The plane-wave cutoff energy was set at 300 eV and the Monkhorst-Pack scheme^41^ was used for the k-point sampling. The optimized lattice parameter a_MnS_ = 5.113 Å, a_PbsS_ = 5.902 Å, and a_MgO_ = 4.237 Å, are very close to the experimental values 5.224 Å, 5.995 Å and 4.220 Å^42^,^43^,^44^.

## Author Contributions

This project was conceived by X.L.M.; TEM experiments were performed by Y.T.Z. and B.Z.; and first principle calculations were carried out by Y.B.X., D.C. and Y.J.W.; all the authors participated in discussion, interpretation of the data, and producing the final version of this paper.
